# *BMC Infectious Diseases* reviewer acknowledgement 2015

**DOI:** 10.1186/s12879-016-1419-0

**Published:** 2016-03-09

**Authors:** Hilary Logan

**Affiliations:** BioMed Central, Floor 6, 236 Gray’s Inn Road, London, WC1X 8HB UK

## Abstract

The editors of *BMC Infectious Diseases* would like to thank all our reviewers who have contributed to the journal in Volume 15 (2015).

**Fernando Abad-Franch**

Brazil

**Stephen Abedon**

USA

**Florence Abravanel**

France

**Muhammad Abubakar**

Pakistan

**Sazaly Abubakar**

Malaysia

**Chad Achenbach**

USA

**Olivia Achonduh-Atijegbe**

Cameroon

**Ishag Adam**

Sudan

**Ayola Akim Adegnika**

Germany

**Javier Afeltra**

Argentina

**Farhat Afrin**

Saudi Arabia

**Rupesh Agrawal**

Singapore

**Golo Ahlenstiel**

Australia

**Hyo Yeong Ahn**

Korea, South

**Aima Ahonkhai**

USA

**Mustapha Ahsaini**

Morocco

**Vineet Ahuja**

India

**Maria Aigner**

Austria

**Marta Aires-De-Sousa**

Portugal

**Katherine Ajdukiewicz**

UK

**Sitara Ajjampur**

India

**MJ Alam**

USA

**Isabelle Albert**

France

**Werner Albrich**

Switzerland

**David Alexander**

Canada

**Vangelis Alexiou**

Greece

**Nahid Ali**

India

**Bachti Alisjahbana**

Indonesia

**Nicolaos Almyroudis**

USA

**Carolyn Alonso**

USA

**Thomas Althaus**

Thailand

**Benjamin Althouse**

USA

**Cosme Alvarado-Esquivel**

Mexico

**Francisco Álvarez García**

Spain

**Carlos Roberto Alves**

Brazil

**Gayatri Amirthalingam**

UK

**Rune Andersson**

Sweden

**Warren Andiman**

USA

**Anuska Andjelkovic Zochowska**

USA

**Anton Andonov**

Canada

**Genevieve Andre-Fontaine**

France

**Nick Andrews**

UK

**Wim Ang**

The Netherlands

**Martin Angelin**

Sweden

**Mehar Angez**

Pakistan

**Francois Angoulvant**

France

**Brian Angus**

UK

**Filippo Ansaldi**

Italy

**Constantine Antonopoulos**

Greece

**Victor H Aquino**

Brazil

**Amber Arain**

Belgium

**Minerva Arce-Fonseca**

Mexico

**Carmen Ardanuy**

Spain

**Katherine Arden**

Australia

**Alicia Arechavala**

Argentina

**Jorge Arevalo**

Brazil

**Emmanuel Arinaitwe**

Uganda

**Frederick Armah**

UK

**George Armah**

Ghana

**Janet Arno**

USA

**Peter H Asdahl**

Denmark

**Elizabeth Ashley**

Thailand

**Lelisa Assebe**

Ethiopia

**Ross Atkinson**

UK

**Enas Attia**

Egypt

**Christian Auer**

Switzerland

**Peter Axelrod**

USA

**Chaw Aye**

Australia

**Andrew Azman**

USA

**Lilian Azzopardi**

Malta

**Subash Babu**

India

**John Baddley**

USA

**Wolfgang Baeumer**

USA

**Nathan Bahr**

USA

**Zhenggang Bai**

China

**Anda Baicus**

Romania

**J Baillie**

UK

**Francis Bajunirwe**

Uganda

**Brook Baker**

USA

**Mirjam Irene Bakker**

The Netherlands

**Manju Bala**

India

**Ludmila Baltazar**

USA

**Alasdair Bamford**

UK

**David Banach**

USA

**Niaz Banaei**

USA

**Ritu Banerjee**

USA

**Pankaj Baral**

USA

**Tristan Barber**

UK

**Dulce Barbosa**

Brazil

**Francis Barin**

France

**Timothy Barkham**

Singapore

**Gavin Barlow**

UK

**Ruanne Barnabas**

USA

**Arvind Baronia**

India

**Naor Bar-Zeev**

Malawi

**Matteo Bassetti**

UK

**Monica Basso**

Italy

**Francisco Bastos**

Brazil

**Matthew Bates**

UK

**Wagner Luiz Batista**

Brazil

**Deepak Batura**

UK

**Abebe Genetu Bayih**

Canada

**Pontius Bayo**

Uganda

**Ken Beagley**

Australia

**Karsten Becker**

Germany

**Soeren Becker**

Germany

**Richard Bedell**

USA

**Rodolfo Begue**

USA

**Pedro Belda-Ferre**

Spain

**Charlotte Bell**

Australia

**Steve Bellan**

USA

**Silvana Belo**

Portugal

**Alexia Belperron**

USA

**Andrea Benedetti**

Canada

**Thomas Bénet**

France

**Abdelouaheb Bennani**

Morocco

**Kim Benschop**

The Netherlands

**Guillaume Béraud**

France

**Bradford Berges**

USA

**Dennis Bergmans**

The Netherlands

**Louis Bernard**

France

**Jose Ignacio Bernardino**

Spain

**Mariolga Berrizbeitia**

Venezuela

**Khalid Beshir**

UK

**Debra Bessen**

USA

**Andrew David Beswick**

UK

**Sarah Bevins**

USA

**Natalie Beylis**

South Africa

**Matthew Beymer**

USA

**N Bhadelia**

USA

**Sanjay Bhagani**

UK

**Preeti Bharaj**

India

**Sandeep Bharaswadikar**

India

**Amrita Bharat**

Canada

**Claver Bhunu**

Zimbabwe

**Sibhatu Biadgilign**

Ethiopia

**Lena Biehl**

Germany

**Marius Birlea**

USA

**Hashem Bishara**

Israel

**Debasis Biswas**

India

**Jason Blackard**

USA

**José-Ramón Blanco**

Spain

**Meridith Blevins**

USA

**Lucie Blok**

The Netherlands

**Frank Bloos**

Germany

**Stijn Blot**

Belgium

**Thomas Bock**

Germany

**Barbara Body**

USA

**Debrah Boeras**

USA

**Almut Böer-Auer**

Germany

**Vicente Boix**

Spain

**Jesper Bonde**

Denmark

**Maryline Bonnet**

Switzerland

**Meatherall Bonnie**

Canada

**Rémy Bonnin**

France

**Celestino Bonura**

Italy

**Andre Boonstra**

The Netherlands

**Timothy Booth**

Canada

**Paola Borella**

Italy

**Vanni Borghi**

Italy

**Philipp Bosshard**

Switzerland

**Julie Bottero**

France

**Valerie Bouchez**

France

**Yap Boum**

Uganda

**Roberto Bovo**

Italy

**Lyudmila Boyanova**

Bulgaria

**Anders Boyd**

France

**Sebastien Boyer**

Madagascar

**Daniel Boyle**

USA

**Nicole Boyle**

USA

**Steven Bradfute**

USA

**Silvia Bressan**

Italy

**Richard Brindle**

UK

**Claire Bristow**

USA

**Murilo Britto**

Brazil

**Etienne Brochot**

France

**Claudia Brodskyn**

Brazil

**Ruth Broering**

Germany

**David Broniatowski**

USA

**Barbara Brown-Elliot**

USA

**Roberta Bruhn**

USA

**Sylvia Bruisten**

The Netherlands

**Fabrice Bruneel**

France

**Vincent Bruno**

USA

**Cheryl Brunton**

New Zealand

**Nele Brusselaers**

Sweden

**Juliet Bryant**

UK

**Udo Buchholz**

Germany

**Maria Jose Buitrago**

Spain

**Ann Burchell**

Canada

**Emily Burger**

Norway

**David Burns**

USA

**Bettina Buttaro**

USA

**Steve Bynon**

USA

**Andrea Maurizio Cabibbe**

Italy

**Omar Caceres**

Peru

**Rui Cai**

The Netherlands

**Morena Caira**

Italy

**Andrea Calcagno**

Italy

**Michael Calderwood**

USA

**Giorgio Calisti**

UK

**Greg Calligaro**

South Africa

**Angela Campbell**

USA

**Adrian Canizalez**

Mexico

**Angela Cannas**

Italy

**Stefania Cannizzo**

Italy

**Jorge Cano**

UK

**Cristina Canteros**

Argentina

**Edmund Capparelli**

USA

**Federico Capparelli**

Argentina

**Luciana Cardoso**

Brazil

**Philip Carling**

USA

**Francesca Carozzi**

Italy

**Fabrice Carrat**

France

**Patrizia Carrieri**

France

**Enitan Carrol**

UK

**Cecilia Godoy Carvalhaes**

Brazil

**Edgar M Carvalho**

Brazil

**Peg Carver**

USA

**Jose Casado**

Spain

**Antonio Cascio**

Italy

**Susan Cassels**

USA

**Antonella Castagna**

Italy

**Sandrine Castelain**

France

**Barbara Castelnuovo**

Uganda

**Maria Catania**

Italy

**Sagnelli Caterina**

Italy

**Francesco Cattel**

Italy

**Serena Cavallero**

Italy

**Peter Cegielski**

USA

**Cornelia Ceianu**

Romania

**Robert Centor**

USA

**Maria Cernada**

Spain

**Gabriela Certad**

France

**Guillaume Chabot-Couture**

USA

**Dave Chadee**

Trinidad and Tobago

**Shua Chai**

USA

**Andrew Chan**

Germany

**Duncan Chanda**

Zambia

**Pranatharthi Chandrasekar**

USA

**Lisa Chang**

Australia

**Rudragouda Channappanavar**

USA

**Francois Chappuis**

Switzerland

**Esmita Charani**

UK

**Patrick Charles**

Australia

**Carmen Charlton**

Canada

**PH Chau**

Hong Kong

**Ruchir Chavada**

Australia

**Porntip Chavalitshewinkoon-Petmitr**

Thailand

**Kalyan Chavda**

USA

**Guilherme Chaves**

Brazil

**Phaikyeong Cheah**

Thailand

**Cynthia Chee**

Singapore

**Chih-Jung Chen**

Taiwan

**Haiying Chen**

China

**Liang Chen**

USA

**Lizhang Chen**

China

**Marcus Chen**

Australia

**Po-Yen Chen**

Taiwan

**Qijun Chen**

China

**Yi-Ming Arthur Chen**

Taiwan

**You-Hua Chen**

Canada

**Zhi-Min Chen**

China

**Pin-Nan Cheng**

Taiwan

**Shih-Lung Cheng**

Taiwan

**Vincent Cheng**

Hong Kong

**Fanny Chereau**

France

**Stephane Chevaliez**

France

**Claire Chewapreecha**

UK

**Jung-Yien Chien**

Taiwan

**Ng Lee Ching**

Singapore

**Nyasha Chin’Ombe**

Zimbabwe

**EM Chisati**

Malawi

**Mohammod Chisti**

Bangladesh

**Yen-Cheng Chiu**

Taiwan

**Nikoloz Chkhartishvili**

Georgia

**Maciej Piotr Chlebicki**

Singapore

**Jun Yong Choi**

South Korea

**Sidharth Chopra**

India

**Teena Chopra**

USA

**Vineet Chopra**

USA

**Chin-Cheng Chou**

Taiwan

**Eric Chow**

Australia

**Abhay Chowdhary**

India

**Abhijit Chowdhury**

India

**Christina Christoffersen**

Denmark

**Helen Chu**

USA

**Hin Chu**

Hong Kong

**Anthony Chua**

Singapore

**Arlene Chua**

Switzerland

**Voo Teck Chuan**

USA

**Abrar Chughtai**

Australia

**Linda Chui**

Canada

**Ju-Young Chung**

South Korea

**Gavin John Churchyard**

South Africa

**Miquel Viñas Ciordia**

Spain

**Ivana Cirkovic**

Serbia

**Mareli Claassens**

South Africa

**Jan Clement**

Belgium

**Gary Clifford**

France

**Nathan Clumeck**

Belgium

**Patrick Coffie**

Côte D’Ivoire

**Adam Cohen**

South Africa

**Bevin Cohen**

USA

**Cheryl Cohen**

South Africa

**Donn Colby**

Viet Nam

**Brenda Coleman**

Canada

**Luis Collado González**

Chile

**Julio Collazos**

Spain

**Paul Collini**

UK

**Arnaldo Colombo**

Brazil

**Philippe Colson**

France

**Martie Conradie**

South Africa

**Carlo Contini**

Italy

**James Conway**

USA

**Alex Cook**

Singapore

**Paul Cook**

USA

**Fiona Cooke**

UK

**Barry Cookson**

USA

**Yacoob Coovadia**

South Africa

**Nicola Coppola**

Italy

**Marli Cordeiro**

Brazil

**Rossana Cordeiro**

Brazil

**Victor Max Corman**

Germany

**Alyssa Cornall**

Australia

**Markus Cornberg**

Germany

**Carlos Costa**

Brazil

**Claudio Costantino**

Italy

**Jean Tenena Coulibaly**

Cote D’Ivoire

**Sonali Coulter**

Australia

**Kevin Couper**

UK

**Benjamin Cowling**

Hong Kong

**Peter Coyle**

UK

**Emanuele Cozzani**

Italy

**Alessandro Cozzi-Lepri**

UK

**Duncan Cranendonk**

The Netherlands

**Olaf L Cremer**

The Netherlands

**Amelieke Cremers**

The Netherlands

**Anne-Claude Crémieux**

France

**Jacob Creswell**

Switzerland

**Pablo Cruces**

Chile

**Nancy Crum-Cianflone**

USA

**Isra Cruz**

Switzerland

**Robert Cunney**

Ireland

**Jessica Cusato**

Italy

**Brian Custer**

USA

**Jeffery Cutter**

Singapore

**Nayle Da Silva**

Brazil

**Prabin Dahal**

UK

**Gunnar Dahlen**

Sweden

**Fredrick Dahlgren**

USA

**Chia-Yen Dai**

Taiwan

**Tine Dalby**

Denmark

**Fortunato D’Ancona**

Italy

**Nick Daneman**

Canada

**Zhisheng Dang**

Japan

**Ina Danquah**

Germany

**Dilip Kumar Das**

New Zealand

**Pranab Das**

UK

**Michael David**

USA

**Christopher Davies**

USA

**D Davis**

USA

**Mohamed Ali Daw**

Libya

**Rod Dawson**

South Africa

**Wu De**

China

**Bertille de Barbeyrac**

France

**Birgitte de Blasio**

Norway

**Thomas de Broucker**

France

**Gabriella De Carli**

Italy

**Elena De Carolis**

Italy

**Daan de Gouw**

USA

**Jokin de Irala**

Spain

**Gennaro De Pascale**

Italy

**Francesco Giuseppe de Rosa**

Italy

**Silvia De Sanjose**

Spain

**Henry de Vries**

The Netherlands

**Jan De Waele**

Belgium

**Chiara De Waure**

Italy

**G Ardine de Wit**

The Netherlands

**Fantahun Defeneh**

Ethiopia

**Abraham Degarege**

Ethiopia

**Takashi Deguchi**

Japan

**Valerio Del Bono**

Italy

**Annarosa Del Mistro**

Germany

**Gilda Maria Barbaro Del Negro**

Brazil

**Claudia Del Vecchio**

Italy

**Cynthia Delgado**

USA

**Casper Den Heijer**

The Netherlands

**Licia Denti**

Italy

**Moutaz Derbala**

Qatar

**Amare Deribew**

Kenya

**T Derrick**

UK

**Benoît Dervaux**

France

**Jean-Claude Desenclos**

France

**Sophie Desmonde**

France

**Roger Detels**

USA

**Gabriella D’Ettorre**

Italy

**Gregor Devine**

Australia

**Ashraf Dewan**

Australia

**Sorabh Dhar**

USA

**Daniel Dhumeaux**

France

**Stefano Di Bella**

Italy

**Antonio Di Biagio**

Italy

**Alvaro Diaz**

Uruguay

**Roland Diel**

Germany

**Matthew Dietz**

USA

**Javier Diez-Domingo**

Spain

**Maria Cecilia Dignani**

Argentina

**Lenie Dijkshoorn**

The Netherlands

**Jo-Anne Dillon**

Canada

**Andrew Dinardo**

USA

**Jodie Dionne-Odom**

USA

**Katharina Diouf**

Germany

**Olgica Djurkovic-Djakovic**

Serbia

**Russell Dlae**

Australia

**Hans-Wilhelm Doerr**

Germany

**Thomas Doker**

USA

**Changzheng Dong**

China

**Thomas Dorlo**

The Netherlands

**Marcus Dorner**

UK

**Horng-Yunn Dou**

Taiwan

**Paul Drain**

USA

**Alison Drake**

USA

**Michèle Dramaix-Wilmet**

Belgium

**Michel Drancourt**

France

**Johannes Dreesman**

Germany

**Robert Dreibelbis**

USA

**Dimitri Drekonja**

USA

**Steven Drews**

Canada

**Nicholas Drey**

UK

**Maria D’Souza**

India

**Mignon Du Plessis**

South Africa

**Nancy Duah**

Ghana

**Raquel Duarte**

Portugal

**Alka Dubey**

India

**Jean-Charles Duclos-Vallée**

France

**Birgitta Duim**

The Netherlands

**Anne Dulioust**

France

**Eileen Dunne**

Australia

**Herve Dupont**

France

**Agnès Dupret-Bories**

France

**Emanuele Durante Mangoni**

Italy

**Solange Durao**

South Africa

**Kerina Duri**

Zimbabwe

**Michael Durkin**

USA

**Malcolm Duthie**

USA

**Richard Dwyer**

Sweden

**Shetty Ravi Dyavar**

USA

**Tim Eckmanns**

Germany

**Obaghe Edeghere**

UK

**Stefan Edlund**

USA

**Kathryn Edwards**

USA

**Simon Edwards**

UK

**Philippe Eggimann**

Switzerland

**Peter Eichacker**

USA

**Lloyd Einsiedel**

Australia

**Koumavi Didier Ekouevi**

Cote D’Ivoire

**Kamal El Bissati**

USA

**Maissa El Raziky**

Egypt

**Johannes Elias**

Germany

**Briony Elliott**

Australia

**George Eluwa**

Nigeria

**Mohammad Emaneinie**

Iran

**Janice Endsley**

USA

**Hock-Liew Eng**

Taiwan

**Mark Enright**

UK

**Olivier Epaulard**

France

**Serpil Erdogan**

Turkey

**Kristine Erlandson**

USA

**Ananias Escalante**

USA

**Janne Estill**

Switzerland

**Concepcion Estivariz**

USA

**Vicente Estrada**

Spain

**Charlesnika Evans**

USA

**Echezona Ezeanolue**

USA

**Massimiliano Fabbiani**

Italy

**Cornelius Faber**

Germany

**Diego Rodrigues Falci**

Brazil

**Vicenç Falcó**

Spain

**Marco Falcone**

Italy

**Joseph Falkinham**

USA

**Oana Falup-Pecurariu**

Romania

**Chi-Tai Fang**

Taiwan

**Maryam Farooqui**

Malaysia

**Lanfranco Fattorini**

Italy

**Saul Faust**

UK

**Jonathan Feelemyer**

USA

**Thomas Fekete**

USA

**Arnaud Fekkar**

France

**Jordan Feld**

Canada

**Jia-Yih Feng**

USA

**Kevin Fennelly**

USA

**Peter Ferenci**

Austria

**Rashida Ferrand**

UK

**Eliane Ferreira**

Brazil

**Pedro Ferreira**

Japan

**James Fielding**

Australia

**Suzanne Filteau**

UK

**Katja Fink**

Singapore

**Ramona Finnie**

USA

**Rebuma Firdessa**

USA

**Peter Fischer**

USA

**Dale Fisher**

Singapore

**Deirdre Fitzgerald-Hughes**

Ireland

**Fidelma Fitzpatrick**

Ireland

**Carolin Fleischmann**

Germany

**Stephanie Fletcher**

Australia

**William Flight**

UK

**Carole Fogg**

UK

**Brian Foley**

USA

**Jason Folster**

USA

**Alex Fonseca**

USA

**Patrice Forget**

Belgium

**Giulio Formoso**

Italy

**Kevin Forsyth**

Australia

**Gregory Fox**

Australia

**Henry S Fraimow**

USA

**Cristiana Franchi**

Italy

**Eduardo Franco**

Canada

**Elisabetta Franco**

Italy

**Simon François**

France

**Daniel Freedberg**

USA

**Matthew Freeman**

USA

**Lucia Freitas**

Brazil

**Hagen Frickmann**

Germany

**Jan Friedrich**

Canada

**Nina Friis-Møller**

Denmark

**Niels Frimodt-Moller**

Denmark

**Holly Frost**

USA

**Chuanxi Fu**

China

**I Fuentes**

Spain

**Keiichi Fujiwara**

Japan

**Francesco Maria Fusco**

Italy

**Giovanni Gabutti**

Italy

**Jacques Gaillat**

France

**Katya Galactionova**

Switzerland

**Shawn Gale**

USA

**Jason Gallagher**

USA

**Chandika Gamage**

India

**Manoj Gambhir**

Australia

**Maria Ganczak**

Poland

**Rita Gander**

USA

**Sumanth Gandra**

India

**Qian Gao**

China

**Maria Garces-Sanchez**

Spain

**Federico Garcia**

Spain

**George Garcia**

USA

**Joao Luis Garcia**

Brazil

**Alberto García-Basteiro**

Mozambique

**Guillermo Garcia-Effron**

Argentina

**Aurora Garcia-Fernandez**

Italy

**Carlo Garzelli**

Italy

**Marie Gauthier**

France

**Philippe Gautret**

France

**Jenn Geddes**

USA

**Larrouy Gerald**

UK

**Dionne Gesink**

Canada

**Antoine Gessain**

France

**Laurent Getaz**

Switzerland

**Corine Geurts Van Kessel**

The Netherlands

**Fatemeh Ghaffarifar**

Iran

**Sarah Gheuens**

USA

**Valeria Ghisetti**

Italy

**Evangelos Giamarellos-Bourboulis**

Greece

**Helen Giamarellou**

Greece

**Cristina Giambi**

Italy

**Ricardo Gianecini**

Argentina

**Antonino Giarratano**

Italy

**Sebastien Gibot**

France

**Jose Pedro Gil**

Sweden

**Brad Gilbertson**

Australia

**Mary Ellen Gilder**

USA

**Francisco Gimenez-Sanchez**

Spain

**Mario Giobbia**

Italy

**Paolo Giorgi Rossi**

Italy

**Delphine Girard**

Switzerland

**Enrico Girardi**

Italy

**Delphine Girlich**

France

**Corrado Girmenia**

Italy

**Sophie Githinji**

Kenya

**Gustavo Giusiano**

Argentina

**Kathryn Glass**

Australia

**Erik Glocker**

Germany

**Cyrille Goarant**

New Caledonia

**Nele Goeyvaerts**

Belgium

**Alejandra Goldman**

Argentina

**Lloyd Goldsamt**

USA

**Edward Goldstein**

USA

**Justyna Gołębiewska**

Poland

**Daniel Golparian**

Sweden

**Andre Gomes**

Brazil

**Jose Gomez-Rial**

Spain

**Cecilia Gonzales-Marin**

UK

**Julià González Martin**

Spain

**Ted Gooley**

USA

**Satish Gopal**

Malawi

**Shubha Gopal**

India

**Gregor Gorkiewicz**

Austria

**Tomasz Gosiewski**

Poland

**Neela Goswami**

USA

**Michihiko Goto**

USA

**Hannelore Gotz**

The Netherlands

**Nelesh Govender**

South Africa

**Charitha Gowda**

USA

**Maira Goytia**

USA

**Dennis Grab**

USA

**Christopher Graber**

USA

**Luigi Gradoni**

Italy

**Morag Graham**

Canada

**Lucia Grandiere-Perez**

France

**James Gray**

UK

**Magdalena Grce**

Croatia

**Jason Grebely**

Australia

**Giampaolo Greco**

USA

**David Greenberg**

USA

**Andrew Greenhill**

Australia

**David Griffith**

USA

**Paul Griffiths**

UK

**Ulla Griffiths**

UK

**Andreas H. Groll**

Germany

**Michelle Jennifer Groome**

South Africa

**Charles Grose**

USA

**Robert Gross**

USA

**Gunnar Hischebeth**

Germany

**Duan Guangcai**

China

**Jeremie Guedj**

France

**Francois Guerin**

France

**Yan Guex-Crosier**

Switzerland

**Thaís Guimarães**

Brazil

**Rajni Gunnala**

USA

**Anuj Gupta**

India

**Ravindra Kumar Gupta**

UK

**Rishein Gupta**

USA

**Shashank Gupta**

USA

**Ankur Gupta-Wright**

UK

**Juan Pablo Gutierrez**

Mexico

**Michael Haber**

USA

**Nagy A. Habib**

UK

**Emilia Hadziyannis**

Greece

**Ferry Hagen**

The Netherlands

**Asrat Hailu**

Ethiopia

**Behzad Hajarizadeh**

Australia

**Mahmoud Halablab**

Lebanon

**Valerie Haley**

USA

**Ronald Hall**

USA

**Sebastian Haller**

Germany

**Gabriel Hamer**

USA

**Richard Hamilton**

USA

**Theodore Hammett**

USA

**Klaus Hamprecht**

Germany

**Camille Hamula**

USA

**Yasmeen Hanifa**

UK

**David Hanna**

USA

**Margaret Hannan**

Ireland

**Ann-Brit Eg Hansen**

Denmark

**Abdul Haque**

Pakistan

**Syed Haque**

India

**Ubydul Haque**

USA

**Pia Hardelid**

UK

**David Harley**

Australia

**Guy Harling**

USA

**P Richard Harrigan**

Canada

**John Harris**

UK

**Hans Hartling**

USA

**Rumina Hasan**

Pakistan

**Zahra Hasan**

Pakistan

**Rodrigo Hasbun**

USA

**Kenji Hashimoto**

Japan

**Angelos Hatzakis**

Greece

**Stephen Hawes**

USA

**Claudia Hawkins**

USA

**Alastair Hay**

UK

**Miao He**

China

**Yu He**

USA

**Pasco Hearn**

Cambodia

**Janneke Heijne**

The Netherlands

**Ghada Helaly**

Egypt

**Gideon Kofi Helegbe**

Ghana

**Markus Hell**

Austria

**Niels Bohse Hendriksen**

Denmark

**Niel Hens**

Belgium

**Melissa Herbst-Kralovetz**

USA

**Sabine Hermans**

Uganda

**Alexandra Hernandez**

USA

**Björn Herrmann**

Sweden

**Jean-Louis Herrmann**

France

**Cord Heuer**

New Zealand

**Kirsty Hewitt**

UK

**Scott Heysell**

USA

**Andrew Hill**

UK

**Itaru Hirai**

Japan

**Kenji Hirayama**

Japan

**Robert Hirt**

UK

**Cedric Hirzel**

Switzerland

**Michele C Hlavsa**

USA

**Jennifer Ho**

Australia

**Paulo Ho**

Brazil

**Nguyen Binh Hoa**

Viet Nam

**Marcia Hobbs**

USA

**Natasha Hochberg**

USA

**Ian Hodgson**

UK

**Boris Hogema**

The Netherlands

**Celia Holland**

Ireland

**David Holland**

USA

**Nusrat Homaira**

USA

**Kam-Lun Ellis Hon**

Hong Kong

**Claire Hooker**

Australia

**Vivian Hope**

UK

**Phil Hopewell**

USA

**Nobuyuki Horita**

Japan

**David Horn**

USA

**Olaf Horstick**

Germany

**Catherine F Houlihan**

UK

**Thomas Allan House**

UK

**Thokozani Hove**

Zimbabwe

**Alison Howarth**

UK

**Michelle Hsiang**

USA

**Ming-Ju Hsieh**

Taiwan

**Chao Wei Hsu**

Taiwan

**Yongfei Hu**

China

**Chiu-Ching Huang**

Taiwan

**Chun-Ta Huang**

Taiwan

**Huang Huang**

USA

**Jee-Fu Huang**

Taiwan

**Shiang-Fen Huang**

Taiwan

**Yen-Tsung Huang**

USA

**Yhu-Chering Huang**

Taiwan

**Yi-Wen Huang**

Taiwan

**Victor Huber**

USA

**Mark Hudak**

USA

**Alan Hudson**

USA

**Li Hui**

China

**Roger Hullin**

Switzerland

**Kristina Hulten**

USA

**Bruce Humphrey**

UK

**Chao-Hung Hung**

Taiwan

**Ivan FN Hung**

Hong Kong

**Ling Lung Hung**

Hong Kong

**Mina Hur**

Korea, South

**Aeron Hurt**

Australia

**Syed Shahzad ul Hussan**

Pakistan

**Wilhelmina Huston**

Australia

**David Hutton**

USA

**Daniela Huzly**

Germany

**Javier Ibáñez**

Spain

**Olubukola Idoko**

Gambia

**Vrettos Ierodiakonou**

Greece

**Ricardo Igreja**

Brazil

**Philip Ind**

UK

**Shingo Inoue**

Japan

**Angela Monica Ionică**

Romania

**Seth Irish**

USA

**Warnow Elon Isaac**

Nigeria

**Nazir Ismail**

South Africa

**Noriko Isobe**

USA

**Cathy Ison**

UK

**Enrique Bernaola Iturbe**

Spain

**Danielle Iuliano**

USA

**Martha Iwamoto**

Uganda

**Collins C Iwuji**

UK

**Anand Iyer**

The Netherlands

**Jacques Izopet**

France

**Kauser Jabeen**

Pakistan

**E Jacobs**

Germany

**Nathalie Jacobs**

Belgium

**Jyotsna Jagai**

USA

**Amita Jain**

India

**Kriti Jain**

USA

**Hamid Jalal**

UK

**Vincent Jamonneau**

France

**Nina Jancar**

Slovenia

**Stephane Jauréguiberry**

France

**Marjan Javanbakht**

USA

**Deborah Jaworski**

USA

**Saroj Jayasinghe**

Sri Lanka

**Seyed Mohammad Jazayeri**

Iran

**Jo Jefferies**

UK

**Wen-Juei Jeng**

Taiwan

**Cheryl Jennings**

USA

**Anders Jensen**

Denmark

**Synne Jenum**

Norway

**Seok Hoon Jeong**

South Korea

**Aaron Jex**

Australia

**Manhong Jia**

China

**Judy Natalia Jiménez**

Colombia

**Fengyi Jin**

Australia

**Akanitt Jittmittraphap**

Thailand

**Anja Joachim**

Austria

**Sophia Johler**

Switzerland

**Reimar Johne**

Germany

**Matthew Johnson**

USA

**James Johnston**

Canada

**Makoto Jones**

USA

**Rachael Jones**

Switzerland

**Silje Bakken Jørgensen**

Norway

**Martin Joyce Brady**

USA

**Justin Julander**

USA

**Anamarija Jurcev Savicevic**

Croatia

**Jessica Justman**

USA

**Prapan Jutavijittum**

Thailand

**S Patrick Kachur**

USA

**Klaus Kaier**

Germany

**Siripen Kalayanarooj**

Thailand

**Natarajaseenivasan Kalimuthusamy**

India

**Andrew Kambugu**

Uganda

**Taro Kamigaki**

Japan

**Witchuda Kamolvit**

Thailand

**Rama Kandasamy**

UK

**Abraham Kandathil**

USA

**Melissa Kang**

Australia

**Chuan-Liang Kao**

Taiwan

**Nathan Kapata**

Zambia

**Arti Kapil**

India

**Melissa Kapulu**

Kenya

**Beatrix Kapusinszky**

USA

**Drosos Karageorgopoulos**

Greece

**Petros Karakousis**

USA

**Panagiotis Karanis**

Germany

**Samuel Kariuki**

Kenya

**Symon**

Kenya

**Maile Karris**

USA

**Panduka Karunanayake**

Sri Lanka

**Desta Kassa**

Ethiopia

**Victoria Katawera**

USA

**Juri Katchanov**

Germany

**Mitsuyo Kawaguchiya**

Japan

**Yoshiiku Kawakami**

Japan

**Uner Kayabas**

Turkey

**Helena Käyhty**

Finland

**Brenda Kazemier**

The Netherlands

**Mary Kearney**

USA

**Tibebeselassie Keflie**

Ethiopia

**Theodoros Kelesidis**

USA

**Christian Keller**

Germany

**David Kelso**

USA

**Brian Kendall**

USA

**Derek Kennedy**

Australia

**Alison Kesson**

Australia

**Yoav Keynan**

Canada

**Ali Khamesipour**

Iran

**Pattara Khamrin**

Thailand

**Muhammad Umair Khan**

Malaysia

**Rafiq Khanani**

Pakistan

**Gulam Khandaker**

Australia

**Ameneh Khatami**

Australia

**Alexander Khoruts**

USA

**Sandra Kik**

The Netherlands

**Kwang-Sik Kim**

USA

**Kyuseok Kim**

South Korea

**Hirokazu Kimura**

Japan

**Rachel King**

Uganda

**Margaret Kingston**

UK

**Agnes Kiragga**

Uganda

**Halid Kirunda**

Uganda

**Pandey Kishor**

Nepal

**Takatoshi Kitazawa**

Japan

**Eili Klein**

USA

**Karin Klingel**

Germany

**Hans-Michael Klinger**

Germany

**Eveline Klinkenberg**

The Netherlands

**Daniela Klobassa**

Austria

**Marisa Klopper**

South Africa

**Tejs Klug**

Denmark

**Jan Kluytmans**

The Netherlands

**Mirjam Knol**

The Netherlands

**Wen-Chien Ko**

Taiwan

**Nobumichi Kobayashi**

Japan

**Marleen Kock**

South Africa

**Sander Koenraadt**

The Netherlands

**Tse Hsien Koh**

China

**Won-Jung Koh**

South Korea

**Alain Kohl**

UK

**Gerjo Kok**

The Netherlands

**Olatunji Kolawole**

South Africa

**Marin Kollef**

USA

**Peter Kolominsky-Rabas**

Germany

**Onn Min Kon**

UK

**Frank Konings**

Philippines

**James Stephen Koopman**

USA

**Neeltje Kootstra**

The Netherlands

**Izabela Korona-Glowniak**

Poland

**Shyam Kottilil**

USA

**Claudia Kozinetz**

USA

**John Kraemer**

USA

**Colleen Kraft**

USA

**Axel Kramer**

Germany

**Ellen Krautkramer**

Germany

**Veit Krenn**

Germany

**Triveni Krishnan**

India

**Shih-Chi Ku**

Taiwan

**Abhay Kudale**

India

**Ayush Kumar**

USA

**Alakes Kumar Kole**

India

**Mark Kuniholm**

USA

**Andreas Kuznik**

USA

**Awewura Kwara**

USA

**Jesse Kwiek**

USA

**Kin On Kwok**

Hong Kong

**Laurence Lachaud**

France

**Philippe Lagacé-Wiens**

Canada

**Jennifer Lai**

USA

**Poh-Chin Lai**

Hong Kong

**Sanjay Lala**

South Africa

**Tommy Tsan-Yuk Lam**

USA

**Tracey Lamb**

USA

**Stephen Lambert**

Australia

**Mohammed Lamorde**

Uganda

**Ruiting Lan**

Australia

**Marcus D Lancé**

The Netherlands

**Caroline Landelle**

France

**Sylvia Laperche**

France

**Kerry LaPlante**

USA

**John A Larbi**

Ghana

**Bryce Larke**

Canada

**Sarah Larney**

Australia

**Cornelia Lass-Floerl**

Austria

**Eric Lau**

Hong Kong

**James Lawler**

USA

**Catherine Lawrence**

UK

**John Lawrenson**

South Africa

**Jen Layden**

USA

**Laura Layland**

Germany

**Christina Lazar**

USA

**Tran-Anh Le**

Viet Nam

**Vincent Le Moing**

France

**Nicole Le Saux**

Canada

**Bee Wah Lee**

Singapore

**Chun Kiat Lee**

Singapore

**Hong Kai Lee**

Singapore

**Ming-Hsun Lee**

Taiwan

**Sang-Won Lee**

South Korea

**Sue Lee**

Thailand

**Todd Lee**

Canada

**Vernon Lee**

Singapore

**Winnie Lee**

Singapore

**Jerome Legoff**

France

**Clara Lehmann**

Germany

**Sebastain Lemmen**

Germany

**Qibin Leng**

China

**Sebastiano Leone**

Italy

**Alexander Lerner**

USA

**Alasdair Leslie**

South Africa

**Victor Leung**

Canada

**Peter Leutscher**

Denmark

**Marilyn Levi**

USA

**Jie Bin Lew**

Australia

**Karl Lewalter**

Germany

**David Lewis**

Australia

**James Lewis**

USA

**Joseph Lewnard**

USA

**George Kam Hop Li**

Hong Kong

**Chen-Yi Liao**

Taiwan

**Qiuyan Liao**

Hong Kong

**Wanqing Liao**

China

**Yun-Fan Liaw**

Taiwan

**Patrick Lillie**

UK

**Jason Limberis**

South Africa

**Wolfgang Lindner**

Germany

**Filippo Lipani**

Italy

**Christopher Lippincott**

USA

**Thiago Lisboa**

Spain

**Margaret Littlejohn**

Australia

**Feng Liu**

USA

**Hsin-Fu Liu**

Taiwan

**Jie Liu**

USA

**Ming-Tsan Liu**

Taiwan

**Sze Hang Kevin Liu**

Hong Kong

**Vivian Liu**

China

**Wei Liu**

China

**Martin Llewellyn**

UK

**Michael Lo**

USA

**Catherine M Loc-Carrillo**

USA

**Nicoletta Locuratolo**

Italy

**Tze Ping Loh**

Singapore

**Elisa Long**

USA

**Richard Long**

Canada

**Yves Longtin**

Canada

**Ana Patricia Lopes**

Portugal

**Guillermo Lopez Campos**

Australia

**Luis Eduardo López-Cortés**

Spain

**Ben Lopman**

UK

**Irene Lorand-Metze**

Brazil

**Jaime Lora-Tamayo**

Spain

**Pierre Loulergue**

France

**Christopher Lowe**

Canada

**Warren Lowman**

South Africa

**Beibei Lu**

USA

**Po-Liang Lu**

Taiwan

**Vivian Luchsinger**

Chile

**Christoph Luebbert**

Germany

**Leo Lui Hong**

Kong

**Robert Lukande**

Uganda

**Aroonlug Lulitanond**

Thailand

**David Lung**

Hong Kong

**Christian Lüring**

Germany

**Irja Lutsar**

Estonia

**Philipp Lutz**

Germany

**Zhiyue Lv**

China

**Dickson Lwetoijera**

Tanzania

**Samantha Lycett**

UK

**David Chien Lye**

Singapore

**G Marshall Lyon**

USA

**Gary Maartens**

South Africa

**Dorothy Machalek**

Australia

**Shingai Machingaidze**

South Africa

**David MacLaren**

Australia

**Gesham Magombedze**

UK

**FMG Magpantay**

USA

**Jagadish Mahanta**

India

**Mohammad Mahmoudi**

Iran

**Matthias Maiwald**

Germany

**Gathsaurie Malavige**

Sri Lanka

**Maricar Malinis**

USA

**Iwona Malinowska**

Poland

**Kylie-Ann Mallitt**

Australia

**Ryan Malosh**

USA

**Monica Malta**

USA

**Helen Maltezou**

Greece

**Caterina Mammina**

Italy

**Si Ming Man**

USA

**Ogenna Manafa**

Ireland

**Niwat Maneekarn**

Thailand

**Jose Ramon Maneiro**

Spain

**Julie Mangino**

USA

**Lisa Manhart**

USA

**Jayanti Mania-Pramanik**

India

**Tobias Manigold**

Switzerland

**Annette Mankertz**

Germany

**Jennifer Manne**

USA

**Oriol Manuel**

Switzerland

**Lamberto Manzoli**

Italy

**Suresh Maray**

Saudi Arabia

**Giulia Marchetti**

Italy

**Andres Mardh**

Sweden

**Jutta Marfurt**

Australia

**Alemka Markotic**

Croatia

**Theodore Marras**

Canada

**Carl Marrs**

USA

**Thomas Marth**

Germany

**Emily Martin**

USA

**Irene Martin**

Canada

**Javier Martin**

UK

**Maria Martin**

Germany

**Richard A Martinello**

USA

**Esteban Martinez**

Spain

**Ignacio Martin-Loeches**

Ireland

**Florian Marx**

Germany

**Emilio Maseda**

Spain

**Mar Masiá**

Spain

**Paul Massion**

Belgium

**Serge Masson**

Italy

**Cesare Massone**

USA

**Claudio Maria**

Mastroianni Italy

**Amy Mathers**

USA

**Catherine Mathews**

UK

**Yasufumi Matsumura**

Japan

**Alberto Matteelli**

Italy

**Dimitrios Matthaiou**

Greece

**Frauke Mattner**

Germany

**Eric Maury**

France

**Harriet Mayanja**

Uganda

**Humphrey Mazigo**

Tanzania

**Wilfred Mbacham**

Cameroon

**Cecilia Mbae**

Kenya

**Anthony Mbonye**

Uganda

**Erasto Vitus Mbugi**

Tanzania

**Sheryl McCurdy**

USA

**Scott McDonald**

The Netherlands

**Fiona McGill**

UK

**Rose McGready**

Thailand

**Skye McGregor**

Australia

**Bradford McGwire**

USA

**Dorian McIlroy**

France

**Mark McKinlay**

USA

**Mary-Louise McLaws**

Australia

**Diane McMahon-Pratt**

USA

**Jim McMenamin**

UK

**Meredith McMorrow**

USA

**Brendan McMullan**

Australia

**Takafira Mduluza**

Zimbabwe

**Jaishri Mehraj**

Germany

**Sanjay Mehta**

USA

**Roberto Melano**

Canada

**Analy Melo**

Brazil

**Francesco Menichetti**

Italy

**Shruti Menon**

Australia

**Andreas Mentis**

Greece

**Andres Merits**

Estonia

**Sharon Meropol**

USA

**Dominik Mertz**

Canada

**Jane Messina**

UK

**David Meya**

Uganda

**Jaimie Meyer**

USA

**Patrick Miailhes**

France

**Janet Midega**

Kenya

**Florence Migot-Nabias**

France

**Benjamin Miko**

USA

**Rafael T Mikolajczyk**

Germany

**Michael Millar**

UK

**Ruth Miller**

Canada

**Richard Milne**

New Zealand

**Cezarina Mindru**

USA

**Corrado Minetti**

UK

**Veenu Minhas**

USA

**Victor Minichiello**

Australia

**Anabela Miranda**

Portugal

**Angelica Miranda**

Brazil

**Veriko Mirtskhulava**

Georgia

**Sharmistha Mishra**

Canada

**Evangelos Misiakos**

Greece

**Carole Mitnick**

USA

**Masashi Mizuguchi**

Japan

**Hala Mohamed**

Egypt

**Vidyarani Mohankumar**

India

**Igor Mokrousov**

Russia

**Monica Molano**

Australia

**Edmund Molesworth**

Australia

**Dinesh Mondal**

Bangladesh

**Francesca Montagnani**

Italy

**Brian Montague**

USA

**Matilde Monteiro-Soares**

Portugal

**Victor Monteon**

Mexico

**Christopher Montgomery**

USA

**Giovanni Montini**

Italy

**Luke Moore**

UK

**Roger Morbey**

UK

**Chantal Morel**

UK

**Frederick Morfaw**

Cameroon

**Mitsuru Mori**

Japan

**Fintan Moriarty**

Switzerland

**Hiroyuki Moriuchi**

Japan

**Carl Morrow**

South Africa

**Joel Mossong**

Luxembourg

**Domenico Motola**

Italy

**Moustafa Mourad**

UK

**Sikhulile Moyo**

Botswana

**Sizulu Moyo**

South Africa

**Sekesai Mtapuri-Zinyowera**

Zimbabwe

**Eric Mueller**

USA

**Lapo Mughini-Gras**

The Netherlands

**Vikramjit Mukherjee**

USA

**JK Mukonzo**

Sweden

**Christiaan Mulder**

The Netherlands

**Matthew Muller**

Canada

**Mathirut Mungthin**

Thailand

**Carmen Munoz-Almagro**

Spain

**David Murdoch**

New Zealand

**Oscar Murillo**

Spain

**Sean Murphy**

USA

**Baba Maiyaki Musa**

Nigeria

**John Muscedere**

Canada

**David Mushatt**

USA

**Doris Mutabazi-Mwesigire**

Uganda

**Nico Mutters**

Germany

**Bernard Naafs**

The Netherlands

**Kesara Na-Bangchang**

Thailand

**Jean Nachega**

USA

**Mathieu Nacher**

French Guiana

**Behzad Nadjm**

Viet Nam

**Y Nagao**

Japan

**Nico Nagelkerke**

The Netherlands

**M Nagl**

Austria

**Cho Naing**

Malaysia

**Vidhya Nair**

Canada

**Vinay Nair**

USA

**Luigi Naldi**

Italy

**Lee Nan-Yao**

Taiwan

**Kanwar Narain**

India

**Edward Nardell**

USA

**Giuseppe Nascetti**

Italy

**Ana Lucia Nascimento**

Brazil

**Cristiana Nascimento-Carvalho**

Brazil

**K Natarajaseenivasan**

India

**Brian Nathanson**

USA

**Sonia Navas-Martin**

USA

**Seema Nayak**

USA

**Godwin Nchinda**

Cameroon

**Momar Ndao**

Canada

**Martial Ndeffo Mbah**

USA

**Dieynaba N’Diaye**

France

**James Neal**

UK

**Jay Neal**

USA

**Keith Neal**

UK

**Peter Neal**

UK

**Issa Nebie**

Burkina Faso

**Kenrad Nelson**

USA

**Mark Nelson**

UK

**Alexander Nemec**

Czech Republic

**Ujjwal Neogi**

Sweden

**Hui-Min Neoh**

Malaysia

**Heinrich Neubauer**

Germany

**Jason Newland**

USA

**Kim Tien Ng**

Malaysia

**Mah-Lee Ng**

Singapore

**Nadia Nguyen**

USA

**Mya Myat Ngwe Tun**

Japan

**Mancini Nicasio**

Italy

**Christos Nicolaides**

USA

**Lindsay Nicolle**

Canada

**Matthias Niedrig**

Germany

**Susanne Dam Nielsen**

Denmark

**Birgit Nikolay**

UK

**Hans Helmut Niller**

Germany

**Chuanyi Ning**

China

**Yuming Ning**

USA

**Ran Nir-Paz**

Israel

**Veeranoot Nissapatorn**

Malaysia

**Rashed Noor**

Bangladesh

**Ingvild Nordøy**

Norway

**Hans Norrgren**

Sweden

**Steven Norris**

USA

**Abigail Norris Turner**

USA

**Niels Nørskov**

Denmark

**Monika Nothacker**

Germany

**Shannon Novosad**

USA

**Norbert Nowotny**

Austria

**Saad Nseir**

France

**Elaine Nsoesie**

USA

**Melissa Nyendak**

USA

**Ali O Mohamed Salem O Boukhary**

Mauritania

**Michael Oberholzer**

USA

**Karina O’Connell**

Ireland

**Catherine O’Connor**

Australia

**Max O’Donnell**

USA

**Onyema Ogbuagu**

USA

**Sung Hee Oh**

South Korea

**Makoto Ohnishi**

Japan

**John Ohoro**

USA

**Iruka Okeke**

Nigeria

**Lucy Okell**

UK

**Sinan Oksuz**

Turkey

**Afiong Oku**

Nigeria

**Kehinde Okunade**

Nigeria

**Sam Okware**

Uganda

**Anna Olsen**

Australia

**Karina Olsen**

Norway

**Yusuf Omosun**

USA

**Geoffrey Omuse**

Kenya

**Raphael Ondondo**

Kenya

**Jason Ong**

Australia

**Gabriel Mbadiwe Onyeama**

Nigeria

**Eline Op De Coul**

The Netherlands

**Paula Opromolla**

Brazil

**Eyal Oren**

USA

**Walter Orenstein**

USA

**Thorsten Orlikowsky**

Germany

**Beatrix Oroszi**

Hungary

**Giovanni Battista Orsi**

Italy

**Enrique Ortega-Gonzalez**

Spain

**Carla Osiowy**

Canada

**Fabio Ostanello**

Italy

**Ronan O’Toole**

Australia

**Akaninyene Otu**

Nigeria

**Kennedy Otwombe**

South Africa

**Mohamed Salem Ould Ahmedou Salem**

Mauritania

**Collins Ouma**

Kenya

**Alexander Outhred**

Australia

**Helieh Oz**

USA

**S Ozkoc**

Turkey

**Marc Paccalin**

France

**Maria Clara Padoveze**

Brazil

**Anne-Laure Page**

France

**Katie Page**

Australia

**John Paget**

The Netherlands

**W Pai**

USA

**Elijah Paintsil**

USA

**Dasja Pajkrt**

The Netherlands

**Andrea Pakula**

USA

**Elizabeth Palavecino**

USA

**Antonio Palazon-Bru**

Spain

**Pedro Palha**

Brazil

**Lucia Pallecchi**

Italy

**Kelli Palmer**

USA

**Domingo Palmero**

Argentina

**Stefan Panaiotov**

Bulgaria

**Aditya K Panda**

India

**Xiao-Li Pang**

Canada

**Annalisa Pantosti**

Italy

**Pasquali Paolo**

Italy

**Vassiliki Papaevangelou**

Greece

**Jesse Papenburg**

Canada

**Manuela Papini**

Italy

**Glaucia Paranhos-Baccalà**

France

**Ramesh Paranjape**

India

**Dimitrios Paraskevis**

Greece

**Raveen Parboosing**

South Africa

**Christopher Parry**

UK

**Fiona Parsonson**

Australia

**Shama Parveen**

India

**Jotam Pasipanodya**

USA

**Tushar B Patil**

India

**John Paul**

UK

**Nicole Pavio**

France

**Androula Pavli**

Greece

**Eric Walter Pefura-Yone**

Cameroon

**Ville Peltola**

Finland

**Ruirui Peng**

China

**Adriana Perez**

USA

**Steven Pergam**

USA

**Guey Chuen Perng**

Taiwan

**Kristina Persson**

Sweden

**Vivekanandan Perumal**

India

**Jonathan Peter**

South Africa

**Linda Petrone**

Italy

**Mariya Petrova**

Belgium

**Michael Pfaller**

USA

**Yvonne Pfeifer**

Germany

**Jody Phelan**

UK

**Warren Phipps**

USA

**Juan Picazo**

Spain

**Russel Pierre**

Jamaica

**Antonio Pignatari**

Brazil

**Benoît Pilmis**

France

**Marcelo Pinto**

Brazil

**Lionel Piroth**

France

**Henry Pleass**

Australia

**Ioannis Pneumatikos**

Greece

**Laura Jean Podewils**

USA

**Jason Pogue**

USA

**Piero Poletti**

Italy

**Chiara Poletto**

France

**Kevin Pollock**

UK

**Larisa Poluektova**

USA

**Virginia Pomar**

Spain

**William Pomat**

Guinea

**Kittiyod Poovorawan**

Thailand

**Isabel Portugal**

Portugal

**Luciano Potena**

Italy

**Lakshmi-Prasad Potluri**

USA

**Stephanie Pouch**

USA

**Anja Poulsen**

Denmark

**Spyros Pournaras**

Greece

**Gerard Pouvourville**

USA

**Koen B Pouwels**

The Netherlands

**Isobel Poynten**

Australia

**Bruno Pozzetto**

France

**Prabda Praphasiri**

Thailand

**Rosa Prato**

Italy

**George Praygod**

Tanzania

**Rebecca Prevots**

USA

**Patricia Priest**

New Zealand

**Miroslav Prucha**

Czech Republic

**Bhupesh Prusty**

Denmark

**Maria Carmen Puertas**

Spain

**Chengli Que**

China

**Mark Quinlivan**

UK

**Michael Quinn**

Australia

**Miguel Quinones-Mateu**

USA

**Yvonne Qvarnstrom**

USA

**Tavs Qvist**

Denmark

**R Negroni**

Argentina

**R Prakash**

India

**Holger F Rabenau**

Germany

**Keith Radcliffe**

UK

**Lewis Radonovich**

USA

**BE Rangaswamy**

India

**Gilles Raguin**

France

**Jaya Rajaiya**

USA

**Anna Ralph**

Australia

**Sanjay Ram**

USA

**Girish Ramachandran**

USA

**Oscar Ramirez**

USA

**Jose Manuel Ramos**

Spain

**Jake Rance**

Australia

**Louise Randall**

Australia

**Tara Randis**

USA

**Merja Rantala**

Finland

**Durga Rao**

India

**Didier Raoult**

France

**Jean-Philippe Rasigade**

France

**Abdolaziz Rastegar Lari**

Iran

**Mehrdad Ravanshad**

Iran

**Henrik Ravn**

Denmark

**G Thomas Ray**

USA

**Pallab Ray**

India

**Jean-Baptiste Rayaisse**

Burkina Faso

**Michael Rayment**

UK

**Phillip Read**

Australia

**Tim Read**

Australia

**Timothy Read**

USA

**Patrick Reading**

Australia

**Gil Redelman-Sidi**

USA

**David Regan**

Australia

**Thomas Reiberger**

Austria

**Utz Reichard**

Germany

**Keli Cristine Reiter**

Brazil

**Lili Ren**

China

**Pilar Retamar**

Spain

**Kelly Reveles**

USA

**Paula Revell**

USA

**Heidi Reynolds**

USA

**Nancy Reynolds**

USA

**Joshua Rhein**

USA

**Peter Rice**

USA

**Vincent Richard**

Senegal

**Riina Richardson**

UK

**Jan Hendrik Richardus**

New Zealand

**Sara Richter**

Italy

**Michaela Riddell**

Australia

**David Riedel**

USA

**Eirini Rigopoulou**

Greece

**Julien Riou**

France

**Ariel Rivas**

USA

**Antonio Rivero**

Spain

**Laura Rivino**

Singapore

**Marco Rizzi**

Italy

**Jennifer Roberts**

Australia

**Rachel Robinson**

USA

**Monica Rocco**

Italy

**Igor Rodrigues**

Brazil

**Sabrina Rodriguez-Campos**

Switzerland

**Benjamin Rogers**

Australia

**Gabriel Rojas-Ponce**

Peru

**Thierry Rolling**

UK

**Nigel Rollins**

South Africa

**William Rom**

UK

**Anne Marie Roque-Afonso**

France

**Nicolas Rose**

France

**Joshua Ross**

Australia

**Sarah Rowland-Jones**

UK

**Patricia Ruas-Madiedo**

Spain

**Franco Maria Ruggeri**

Italy

**Diego Ruiz Moreno**

USA

**Heiner Ruschulte**

Germany

**Fiona Russell**

Australia

**George Rutherford**

USA

**Eva Ruzic-Sabljic**

Slovenia

**Magda Rybicka**

Poland

**Shiva S Halli**

Canada

**Saraswathy Sabanathan**

Viet Nam

**Francesca Sabbatini**

Italy

**Carla Sabia**

Italy

**Caroline Sabin**

UK

**Lora Sabin**

USA

**Karuna Sagili**

India

**Bibhuti Saha**

India

**Jitendra Sahu**

India

**Akihiko Saitoh**

Japan

**Eduardo Salazar-Lindo**

Peru

**Max Salfinger**

USA

**Cassandra Salgado**

USA

**Faouzy Saliba**

France

**Fernando Salvador**

Spain

**Nadia Sam-Agudu**

Nigeria

**Taraz Samandari**

USA

**Banu Sancak**

Turkey

**Susan Sanchez**

USA

**Alexandra Sánchez**

Brazil

**Naveen Sankhyan**

India

**Pitak Santanirand**

Thailand

**Cristina Santos**

Portugal

**Daniel Santos**

Brazil

**Jose Ramon Santos**

Spain

**Roberto Santos**

USA

**Rajiv Sarkar**

India

**Bahador Sarkari**

Iran

**Manoj Sarma**

USA

**Joe Sasadeusz**

Australia

**Michael Satlin**

USA

**Sarah Satola**

USA

**Catherine Satzke**

Australia

**Andreas Sauerbrei**

Germany

**John Saunders**

UK

**Frank Scannapieco**

USA

**Simon Schaaf**

South Africa

**Frieder Schaumburg**

Germany

**Simone Scheithauer**

Germany

**Andre Scherag**

Germany

**Consuelo Schiroli**

Italy

**Susanne Schmitz**

Ireland

**Kathryn Schnippel**

South Africa

**Henrik Carl Schønheyder**

Denmark

**Pieter Schreuder**

The Netherlands

**Constance Schultsz**

The Netherlands

**Lucas Schulz**

USA

**Jörg Schüpbach**

Switzerland

**Andrew Schwaderer**

USA

**William Schwan**

USA

**Jeremy Schwartz**

USA

**John Scott**

USA

**Eric Seaberg**

USA

**Andrew Seaton**

UK

**Ludwig Sedlacek**

Germany

**Gad Segal**

Israel

**Edelwisa Segubre-Mercado**

Philippines

**Marva Seifert**

USA

**Moorine Penninah Sekadde**

Uganda

**Aggrey Semeere**

Uganda

**Joseph Sempa**

Uganda

**Chaminda Jayampath Seneviratne**

Singapore

**Piseth Seng**

France

**Elisavet Serti**

USA

**Chetan Seshadri**

USA

**Ajay Sethi**

USA

**Alberto Severini**

Canada

**Fiona Shackley**

UK

**Lena Shah**

Canada

**Nipam Shah**

USA

**Shohreh Shahmahmoodi**

Iran

**Laura Shallcross**

UK

**Isdore Chola Shamputa**

Canada

**Masoomeh Shams-Ghahfarokhi**

Iran

**Arun Sharma**

India

**Pragya Sharma**

USA

**Kirill Sharshov**

Russia

**Kimberly Shea**

USA

**Xuzhuang Shen**

China

**Edward Sherwood**

USA

**Veena Shetty**

India

**Jim Sheu**

Taiwan

**Marcia Shew**

USA

**Hiroyuki Shimizu**

Japan

**Sourya Shrestha**

USA

**Chin-Chung Shu**

Taiwan

**P Shuetz**

Switzerland

**Dhaval Shukla**

India

**Jennifer Shuldiner**

Israel

**Emily Sickbert-Bennett**

USA

**Scott Sieg**

USA

**Ilias Siempos**

Greece

**Sheetal Silal**

South Africa

**Nozza Silvia**

Italy

**Bryony Simmons**

UK

**Victoria Simms**

UK

**Patricia Simner**

USA

**Itu Singh**

India

**Leung Kei Siu**

Taiwan

**Girts Skenders**

Latvia

**Donald Skinner**

South Africa

**Tina Skinner-Adams**

Australia

**Lars Skog**

Sweden

**David Skurnik**

USA

**Miha Skvarc**

Slovenia

**Emma Slaymaker**

UK

**Robert Slinger**

Canada

**Derek Sloan**

Malawi

**Hans-Christian Slotved**

Denmark

**Colette Smit**

The Netherlands

**Anja Smith**

South Africa

**Kellie Smith**

USA

**Stephen Smith**

Ireland

**Alex Smithson**

Spain

**Davida Smyth**

USA

**René Snacken**

Sweden

**Li-Hwei Sng**

Singapore

**Evan Snitkin**

USA

**Kathryn Snow**

Australia

**Nidhi Sofat**

UK

**RS Solomons**

Zambia

**Mark Sonderup**

South Africa

**Manish Soneja**

India

**Elijah Songok**

Kenya

**Geoffrey Sonn**

USA

**Pam Sonnenberg**

UK

**Geeta Sood**

USA

**Seema Sood**

India

**Antoni Soriano-Arandes**

Spain

**Timo Sorsa**

Finland

**Nestor Sosa**

Panama

**Ana E Sousa**

Portugal

**Neila Souza**

Brazil

**D Sow**

France

**Vincenzo Spagnuolo**

Italy

**Lodewijk Spanjaard**

The Netherlands

**Iris Spiliopoulou**

Greece

**Sandra Springer**

USA

**Nicola Squillace**

Italy

**Chandrashekhar Sreeramareddy**

Nepal

**Saranya Sridhar**

UK

**Willy Ssengooba**

Uganda

**Janneke Stalenhoef**

The Netherlands

**Cecilia Stålsby Lundborg**

Sweden

**Lola Stamm**

USA

**Carol Stanciu**

Romania

**Miles Stanford**

UK

**Catherine Stein**

USA

**Ellen Stein**

USA

**Mart L Stein**

The Netherlands

**Joerg Steinmann**

Germany

**Peter Steinmann**

Switzerland

**Niamh Stephenson**

Australia

**Kasia Stepniewska**

UK

**Erin Stern**

South Africa

**Vanessa Stevens**

USA

**Andrew J Stewardson**

Australia

**Benoit Stijlemans**

USA

**Ellen Stobberingh**

The Netherlands

**Nicole Stoesser**

UK

**Jason Stout**

USA

**Kristoffer Strålin**

Sweden

**Andrea Streng**

Germany

**Klemen Strle**

USA

**Birgit Strommenger**

Germany

**Carol Strong**

Taiwan

**Judith Strymish**

USA

**Chien-Wei Su**

Taiwan

**Arnold Suda**

Germany

**Staci Sudenga**

USA

**Jonathan D Sugimoto**

USA

**Barbara Suligoi**

Italy

**Giorgia Sulis**

Italy

**David Sullivan**

USA

**Ayako Sumi**

Japan

**Ping Sun**

USA

**Yan Sun**

Singapore

**Lavanya Suneetha**

India

**Michael Surette**

Canada

**Johari Surin**

Malaysia

**Faisal Syed**

USA

**Piotr Szweda**

Poland

**Alexis Tabah**

Australia

**Saadia Tabassum**

Pakistan

**Sepehr Tabrizi**

Australia

**Terence Tafatatha**

UK

**Patrick Taffé**

Switzerland

**Sarah Taimur**

USA

**Ai Takano**

Japan

**Antoine Talarmin**

France

**Banhock Tan**

Singapore

**Kit Mun Tan**

Malaysia

**Litjen (LJ) Tan**

USA

**Thuan-Tong Tan**

Singapore

**Tina Tan**

USA

**Atsuchi Tanaka**

Japan

**Mariza Tancredi**

Brazil

**Aaron Tande**

USA

**Julian Wei Tze Tang**

UK

**Min Moon Tang**

Malaysia

**Weiming Tang**

China

**Weiming Tang**

USA

**Yi-Wei Tang**

USA

**Meltem Tasbakan**

Turkey

**Carlo Tascini**

Italy

**Nancy Tee**

Singapore

**Felipe Teixeira De Mello Freitas**

Brazil

**Fasil Tekola-Ayele**

USA

**Raymond Tellier**

Canada

**John Tembo**

China

**David Templeton**

Australia

**Gabriel Tender**

USA

**Christine Teng**

Singapore

**Masanori Terajima**

USA

**Dianne (Anja) Terlouw**

UK

**Anders Ternhag**

Sweden

**Vanessa Terra**

UK

**Eyasu Teshale**

USA

**Raymond Tetteh**

Ghana

**Elli Theel**

USA

**Robert Thimme**

Germany

**Matthew Thoendel**

USA

**Corinne Thompson**

Viet Nam

**Kimberly Thompson**

USA

**Emily Thorell**

USA

**Guy Thwaites**

Viet Nam

**Huai-Yu Tian**

China

**Ellen Tijsse-Klasen**

The Netherlands

**Marcello Tirani**

Italy

**Saruda Tiwananthagorn**

Thailand

**Dileep Tiwari**

South Africa

**Michele Tizzoni**

Italy

**Kelvin To**

Hong Kong

**Nguyen Linh Toan**

Viet Nam

**Selma Tobudic**

Austria

**Viktorija Tomic**

Slovenia

**Steven Tong**

Australia

**Estee Torok**

USA

**Kwasi Torpey**

Nigeria

**Egídio Torrado**

Portugal

**Francesco Torre**

Italy

**Antoni Torres**

Spain

**Anna Maria Tortorano**

Italy

**Grazia Tosone**

Italy

**Patricia Totten**

UK

**Chafia Touil-Boukoffa**

Algeria

**Katy Town**

UK

**Andrea Tramarin**

Italy

**Barbara Trautner**

USA

**Elina Trembizki**

Australia

**Mariangela Trindade**

Brazil

**Quynh Mai Trinh**

Viet Nam

**Birger Trollfors**

Sweden

**Marius Trøseid**

Norway

**Stephanie Troy**

USA

**Allan Truant**

USA

**Cecilia Trucchi**

Italy

**Feng-Jen Tsai**

Taiwan

**Eiirni Tsakiridou**

Greece

**Herman Tse**

Hong Kong

**Wondewosen Tsegaye**

Ethiopia

**Kuo-Chih Tseng**

Taiwan, Republic Of China

**Maria Tsolia**

Greece

**Vivien Tsu**

USA

**Edouard Tuaillon**

France

**Joseph Tucker**

China

**Susan Tuddenham**

USA

**Carrie Tudor**

USA

**Christine Turenne**

Canada

**Joanne Turner**

USA

**Paul Turner**

Cambodia

**Robin Turner**

Australia

**Ombretta Turriziani**

Italy

**Jimmy Twin**

Australia

**Shahadat Uddin**

Australia

**Kingsley Nnanna Ukwaja**

Nigeria

**Noman Ul Haq**

Pakistan

**Kristen Underhill**

USA

**AM Upfill-Brown**

USA

**Vytautas Usonis**

Lithuania

**Louis Valiquette**

Canada

**Andrew Vallely**

Australia

**Aarthy Vallur**

USA

**Florent Valour**

France

**Marita van de Laar**

USA

**Gerd van der Auwera**

Belgium

**Menno van der Eerden**

The Netherlands

**Tonja van der Kuyl**

The Netherlands

**Mark van der Linden**

Germany

**Nicoline van der Maas**

The Netherlands

**Marianne AB van der Sande**

The Netherlands

**GD van der Spuy**

South Africa

**Martie van der Walt**

South Africa

**H Rogier van Doorn**

Viet Nam

**David van Duin**

USA

**AM van Furth**

The Netherlands

**Caroline van Gemert**

Australia

**Martijn van Griensven**

Germany

**Nelda van Soelen**

South Africa

**Ronald van Toorn**

South Africa

**G Van Well**

The Netherlands

**Bias Vandack**

Brazil

**Dana Vanlandingham**

USA

**Anna H van’t Hoog**

The Netherlands

**Gilles Vanwalleghem**

Belgium

**Sophie Vanwambeke**

Belgium

**Anne Vardo-Zalik**

USA

**Daniel Vitor Vasconcelos Santos**

Brazil

**Vipin M Vashishtha**

India

**Shawn Vasoo**

Singapore

**María Velasco**

Spain

**Mario Venditti**

Italy

**Delphis Vera**

Peru

**S Vergara**

Spain

**Paul Verhoeven**

France

**Neena Verma**

India

**Eva Veronesi**

Switzerland

**Florens GA Versteegh**

The Netherlands

**Evelien Verstraete**

Belgium

**Suzanne Verver**

The Netherlands

**Jaco J Verweij**

The Netherlands

**Frédéric Veyrier**

Canada

**Diego Viasus**

Colombia

**David Vickers**

Canada

**Ivan Vickers**

Jamaica

**Roberto Vidal**

Chile

**Margarida Vigário**

Portugal

**Tara Vijayan**

USA

**Dhanasekaran Vijaykrishna**

Singapore

**Miguel Vinas**

Spain

**Manfrin Vinicio**

Italy

**Christopher Vinnard**

USA

**DH Visser**

The Netherlands

**Marianne Visser**

South Africa

**Matteo Vitali**

Italy

**Lenka Vodstrcil**

Australia

**Martin Vogel**

Germany

**Antonio Volpi**

Italy

**Andrea Von Groll**

Brazil

**Claire Von Mollendorf**

South Africa

**Lutz Von Müller**

Germany

**Ann Vossen**

The Netherlands

**Lung Vu**

USA

**Tytti Vuorinen**

Finland

**Jeannette Wadula**

South Africa

**Florian Martin Erich Wagenlehner**

Germany

**Jesse Waggoner**

USA

**Alpana Waghmare**

USA

**Helga Wagner**

Austria

**Catriona Waitt**

UK

**Tim Walker**

Rwanda

**Elisabetta Walters**

South Africa

**Jann-Tay Wang**

Taiwan

**Jann-Yuan Wang**

Taiwan

**Jinfeng Wang**

China

**Lin Wang**

Taiwan

**Maggie Haitian Wang**

Hong Kong

**Sheng-Fan Wang**

Taiwan

**Tao Wang**

China

**Deborah Ward**

UK

**Nicola Wardrop**

UK

**David Wareham**

UK

**Adilia Warris**

UK

**Gregory Waryasz**

USA

**Edward Waters**

Australia

**Laura Waters**

UK

**Chand Wattal**

India

**Rachel Wattier**

USA

**Andy Wearn**

New Zealand

**Adam Weber**

USA

**Heinrich Weber**

Australia

**Scott Weese**

Canada

**Junni Wei**

China

**Louis Weiss**

USA

**Scott Weiss**

USA

**Christian Wejse**

Denmark

**Christina Welinder-Olsson**

Sweden

**Xi Wen**

USA

**Nicolas Wentzensen**

USA

**Lars Westblade**

USA

**Dawn Wetzel**

USA

**Jaclyn White**

USA

**Nathan White**

USA

**Andrew Christopher Whitelaw**

South Africa

**Timothy Wiemken**

USA

**Annelies Wilder-Smith**

Singapore

**Katalin Wilkinson**

South Africa

**Birgit Willinger**

Austria

**Matthias Willmann**

Germany

**Peter Wilson**

UK

**John Wingard**

USA

**Brita Askeland Winje**

Norway

**Andrea Winkler**

Australia

**Kevin Winthrop**

USA

**Carl Heinz Wirsing Von Koenig**

Germany

**Desalegn Woldeyohannes**

Ethiopia

**Hilary Wolf**

USA

**Elke Wollants**

Belgium

**Nicole Wolter**

South Africa

**Karen Wong**

USA

**Ngai Sze Wong**

Hong Kong

**Christopher Wood**

USA

**Darcy Wooten**

USA

**Dan Wootton**

UK

**Michael Worobey**

USA

**David Wright**

UK

**M Wroblewska**

Poland

**Bin Wu**

China

**David Bin-Chia Wu**

Taiwan

**Lawrence Shih Hsin Wu**

Taiwan

**Ming-Shiang Wu**

Taiwan

**Peng Wu**

Hong Kong

**Lihua Xiao**

USA

**Xianhong Xie**

USA

**Zheng Xing**

USA

**Biao Xu**

China

**Howard Xu**

USA

**Kari Yacisin**

USA

**Laith Yakob**

UK

**Jie Yan**

China

**Yongping Yan**

China

**Frances Yang**

USA

**Samuel Yang**

USA

**Stephanie Yanow**

Canada

**Cedric Yansouni**

Canada

**Satomi Yara**

Japan

**Masaki Yasukawa**

Japan

**Michio Yasunami**

Japan

**Tom Yates**

UK

**Barbara Yawn**

USA

**Kojo Yeboah-Antwi**

USA

**Venkat Laxmi Yeruva**

USA

**Ahmet Yılmaz Çoban**

Turkey

**Jae-Joon Yim**

South Korea

**Cyril Yip**

Hong Kong

**Jean Cyr Yombi**

Belgium

**Lay-Myint Yoshida**

Japan

**Barnaby Young**

Singapore

**Jo-Anne Young**

USA

**Kung-Chia Young**

Taiwan

**Zobair Younossi**

USA

**Chong-Jen Yu**

Taiwan

**Luo-Ting Yu**

China

**Chee Fu Yung**

Singapore

**Hans L Zaaijer**

The Netherlands

**Mauro Zaccarelli**

Italy

**Keivan Zandi**

Malaysia

**Isabella Zanella**

Italy

**Maurizio Zazzi**

Italy

**Jonathan Zelner**

USA

**Erliang Zeng**

USA

**Nicola Zetola**

Botswana

**Chiyu Zhang**

China

**Di Zhang**

USA

**Jun Zhang**

China

**Yanan Zhao**

USA

**Pingyu Zhou**

China

**Yanhe Zhou**

China

**Xing-Quan Zhu**

China

**Yefei Zhu**

China

**Panayiotis Ziakas**

USA

**David Zielinski**

Canada

**Hisham Ziglam**

UK

**Marya Zilberberg**

USA

**Daniel Zinder**

USA

**Walter Zingg**

Switzerland

**York Zoellner**

Germany

**Huachun Zou**

Australia

**Benjamin Zuber**

France

**Simbarashe Peter Zvada**

South Africa

**Alice Zwerling**

USA

